# Diffusion Analyses along Mean and Gaussian-Curved Membranes with CurD

**DOI:** 10.1021/acs.jpclett.4c00338

**Published:** 2024-03-14

**Authors:** Balázs Fábián, Matti Javanainen

**Affiliations:** † Institute of Organic Chemistry and Biochemistry of the Czech Academy of Sciences, Flemingovo nám. 542/2, CZ-16000 Prague 6, Czech Republic; ¶ Institute of Biotechnology, University of Helsinki, FI-00790 Helsinki, Finland

## Abstract

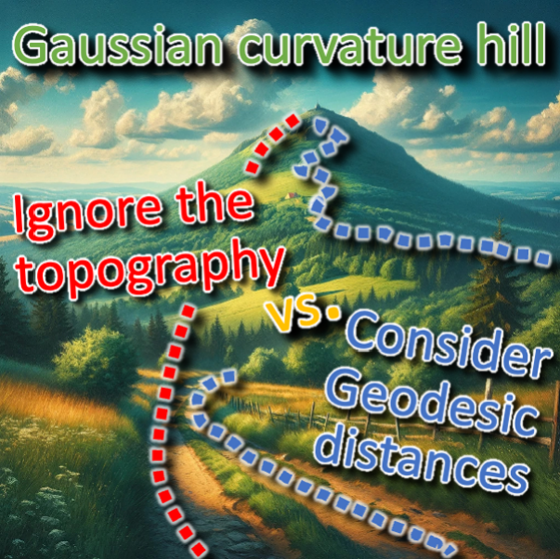

Curved cellular membranes are both abundant and functionally relevant. While novel tomography approaches reveal the structural details of curved membranes, their dynamics pose an experimental challenge. Curvature especially affects the diffusion of lipids and macromolecules, yet neither experiments nor continuum models distinguish geometric effects from those caused by curvature-induced changes in membrane properties. Molecular simulations could excel here, yet despite community interest toward curved membranes, tools for their analysis are still lacking. Here, we satisfy this demand by introducing CurD, our novel and openly available implementation of the Vertex-oriented Triangle Propagation algorithm to the study of lipid diffusion along membranes with mean and/or Gaussian curvature. This approach, aided by our highly optimized implementation, computes geodetic distances significantly faster than conventional implementations of path-finding algorithms. Our tool, applied to coarse-grained simulations, allows for the first time the analysis of curvature effects on diffusion at size scales relevant to physiological processes such as endocytosis. Our analyses with different membrane geometries reveal that Gaussian curvature plays a surprisingly small role on lipid motion, whereas mean curvature; i.e., the packing of lipid headgroups largely dictates their mobility.

State-of-the-art molecular simulations have reached physiologically relevant length or time scales,^1−6^ and the next challenge is to visit both frontiers in a single simulation. Notably, many key functions related to these membranes encapsulating either the entire cell or its organelles involve significant local membrane curvature.^7^ The plasma membrane has specific invaginated signaling platforms,^8^ the complex and dynamic topography of the mitochondrial inner membrane is involved in numerous biological processes,^9^ and the cells store energy in lipid droplets that bud into the cytosol from the membrane surrounding the endoplasmic reticulum.^10^ In these processes, curvature is generated by both lipids^11^ and proteins.^12^ Moreover, they can both also sense curvature, leading to spatial sorting with functional implications.^13,14^

Adequate modeling^15,16^ and analysis^17−20^ of such nonplanar lipid bilayers requires the development of algorithms that take into account the geometry of the membrane.^6^ A particularly fascinating property impacted by membrane curvature is the lateral diffusion of membrane components, which has a direct influence on the interpretation of experimental results obtained from techniques such as Fluorescence Correlation Spectroscopy (FCS) and Single Particle Tracking (SPT) that often assume planar geometries.^21−23^ Notably, the effects of the underlying curvature can be falsely interpreted as slowed-down or even anomalous diffusion,^23−25^ calling for further clarification by alternative approaches such as computer simulations.

Biological membranes undergo thermal fluctuations, affecting their curvature. These fluctuations are generally considered to decrease the experimentally observed mobility by increasing the membrane thickness or extending the geometric path.^26^ Taking the dynamic membrane fluctuations into account would require the treatment of changing surfaces.^27^ Instead, here, we restrain ourselves to work in the limit of static membrane shape,^28^ which is an excellent approximation for endocytic vesicles and structures such as the cristae in the endoplasmic reticulum (ER) or auditory outer hair cells.^29,30^ Moreover, even membranes undergoing endocytic budding on the time scale of minutes seem static for lipids diffusing across their typical size in a matter of milliseconds. Another aspect of the classification of membrane surfaces is their curvature. The curvature of a surface can be completely described by two scalar fields, the Gaussian curvature *K*(**r**) and the mean curvature *H*(**r**). Caveolae and budding vesicles with nonzero Gaussian curvature have radii of a few dozen to a hundred nanometers.^31^ Tubular structures such as those in the mitochondrion are examples of developable surfaces; that is, they only have nonvanishing mean curvature.^32^ The ER is also of great interest due to its complex membrane topology of folded membranes with highly curved regions and due to being the site of lipid droplet biogenesis.^33−35^

Several studies employing continuum methods have investigated the effect of both mean and Gaussian curvature on biological membranes,^28,36−38^ resulting in two major conclusions. First, at the continuum level, the surface mobility of particles can depend only on Gaussian curvature *K* and not on mean curvature *H*. This is related to the fact that developable surfaces—surfaces with vanishing *K*—are isometric to a plane.^36^ Obviously, this is true only for diffusion along the surface, whereas any curvature, *K* or *H*, will affect the observed motion of the particle in experiments that assume a planar membrane. Second, on surface regions with *K* > 0 (elliptic paraboloids) mobility is thought to decrease, while on region with *K* < 0 (hyperbolic paraboloids) it is increased.^36,37^ In addition to these observations, the ratio of the real and projected long-time diffusion coefficients are shown to closely follow a so-called area-scaling law^28^ under a broad range of conditions. Details about the area-scaling law can be found in the Supporting Information (section 3.3).

A general drawback of mesoscale simulations of elastic membrane models is their inability to account for nonflat free-energy landscapes due to lipid packing effects, or the inclusion of proteins or other membrane heterogeneities. These effects carry the possibility of mean curvature influencing surface diffusion through heterogeneous membrane structures.^38^ While such effects are explicitly inherent in molecular simulations, their analyses present another kind of challenge. Namely, the majority of the current analysis tools fall short of dealing with the changing membrane normal in curved membranes.^39^ Another issue is that in Gaussian-curved membranes fairly complex algorithms are required for the calculation of the shortest distances along the curved surface (geodesics), yet these geodesics are essential for any diffusion analyses. Unfortunately, no currently available tools for the analysis of biomembrane simulations implement such algorithms, and hence, the relationships between membrane curvature, lipid packing, and lateral diffusion remain unresolved.

Here, we fill this fundamental gap by implementing, adapting, and optimizing an existing algorithm from another field to analyze lipid motion. Our software, coined CurD, allows for the first time the calculation of Mean Square Displacement (MSD) along curved membranes from trajectories generated using molecular simulations and thus provides novel insights into the curvature dependencies of lipid diffusion. We apply the method to coarse-grained Martini 2^40,41^ simulations of phospholipid bilayers forming a vesicular bud-like membrane protrusion (“Budded”) and an undulating wave-like surface (“Wave”). For the complete list of simulated systems, see section 1 of the Supporting Information. Although slightly overemphasized, the mean and Gaussian curvatures of our simulated systems are not far from those present in some biological systems. All relevant details of the simulations can be found in section 2 of the Supporting Information. The simulated systems and their curvatures are listed in Figure 1. The membrane in the “Wave” system has only mean curvature, *H*, and hence it is isomorphic to a plane. This is not true for the lipid bilayer in the “Budded” system, as it also possesses a Gaussian curvature, *K*. An important attribute of Gaussian curvature is its insensitivity to the orientation of the constituents (lipids or membrane proteins) of the bilayer. A bowl-shaped (saddle-shaped) region always has positive (negative) Gaussian curvature, irrespective of whether the membrane bulges into the cytoplasmic or the extracellular space (and similarly for membranes other than the plasma membrane). The curvatures (bottom panels of Figure 1), while properly capturing the topology of the system, exhibit some irregularities due to the use of mesh surfaces (see section 3 the Supporting Information). Even though their relative sign depends on the chosen leaflet, a strong correlation between the magnitudes of *H* and *K* is clearly apparent.

**Figure 1:**
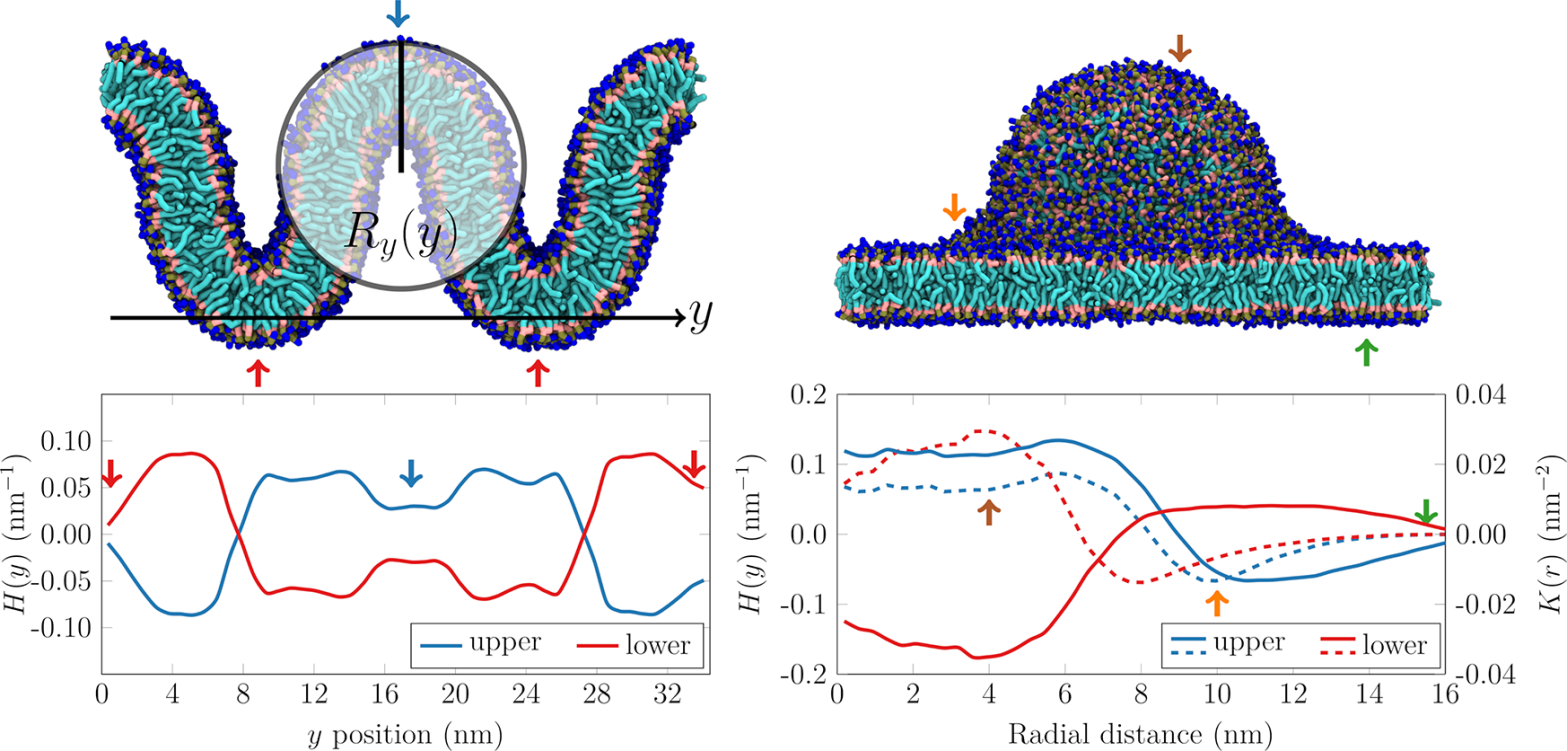
Top: Snapshots of the simulated “Wave” (left) and “Budded” (right) systems. Bottom: Average mean (*H*, smooth lines) and Gaussian (*K*, dashed lines) curvatures as a function of the position along the *y* axis of the “Wave” and the distance from the center of the bud, respectively. Blue line: upper leaflet. Red line: lower leaflet. The leaflet normal vector was always taken to point from the acyl chains toward the headgroups. The arrows indicate regions of a given curvature. For a developable surface such as the “Wave” system, *H*(*y*) = (2*R*_*y*_(*y*))^−1^ is the inverse of the diameter of the *osculating cylinder* of radius *R*_*y*_(*y*) to the surface at the corresponding value of *y*.

The actual displacements of particles constrained to move on a surface are accurately described using geodetic distances *d*(**r**_*i*_(*t*_2_), **r**_*i*_(*t*_1_)),^36,37^ where **r**_*i*_(*t*) denotes the position of the *i*^th^ particle at time *t*. Geodetic distances can be efficiently computed on mesh surfaces using specialized methods, even without explicitly storing or constructing the corresponding shortest path.^42^ One such algorithm is the relatively recent Vertex-oriented Triangle Propagation (VTP) of Qin et al.^43^ that simultaneously computes all distances from a source vertex to all other vertices on the mesh. Therefore, as the first step of our approach, we created a triangular mesh surface from each of the leaflets of the simulated membranes, using the center of mass of the lipid as a proxy for the center of diffusion. For the generation of the mesh, we used our custom code. Then, we used the position of the closest vertex as an approximation of the true surface position of the lipid center of mass. For sufficiently fine meshes, the error introduced by this discretization is negligible, as shown in section 3 of the Supporting Information. To make full use of the VTP algorithm and avoid multiple evaluation of the individual source vertices, we invented a scheme where we group the displacements in a particular way. Importantly, for any kind of MSD evaluation, one needs to know the initial and final positions of the diffusing particle along with the time taken to cover the path, also known as *lag time*, denoted by Δ. We write triplets of integers (start vertex, end vertex, and lag time) representing the paths of particles to a binary file and suitably sort these triplets. By performing the sorting, all paths starting or ending at a given vertex form a contiguous block, irrespective of the lag time. Then, a single call of the VTP algorithm on the source vertex *i* is sufficient to evaluate one complete block, as all of the paths that start or end at vertex *i* are grouped together, independently of the lag time. Finally, we assign the calculated geodetic distances to the source and end vertices of the path, while keeping track of the lag time. Once properly processed, the mesh contains the discretized spatial distributions of the geodetic MSD (gMSD) values at various lag times. Importantly, our approach is flexible enough to handle surface meshes of arbitrary shapes^2,18^ subject to the condition that they do not change in time. It is also modular, so that the distance calculation algorithm can readily be swapped with other existing methods. An illustration of the major steps of our algorithm is presented in Figure 2. Details on the creation of the surface meshes, the magnitude of the discretization error, the handling of periodic boundary conditions, and information about the scaling of the algorithm can be found in the Supporting Information. Here, we used meshes of 40,000 points with a spacing of ≈0.4 nm to cover four periodic images, yet the algorithm scales reasonably well to meshes at least twice this size, corresponding to membranes with over 10,000 lipids. Independent calls of the VTP algorithm for different source vertices allow for a further increase in studied system size through the implemented trivial parallelization. For the sake of simplicity, the computed gMSD curves are interpreted by assuming a free diffusion model at each point of the surface, *D*_geo_ = gMSD/4Δ. The free diffusion model neglects the appearance of drift terms due to the nonflat free energy landscape,^44^ that is, the nonuniform lipid density. However, the inclusion of these effects is beyond the scope of the present work.

**Figure 2:**
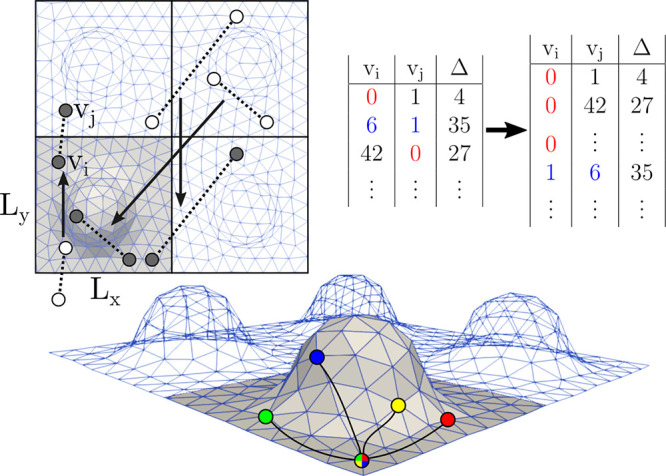
Illustration of the main steps of calculating the geodetic Mean Square Displacements. After surface meshing, all *particle displacements*, that is, initial and final particle positions (white circles) separated by a lag time, Δ, are mapped onto the surface, ensuring that both the starting and end points are on the mesh, as close to the origin as possible (gray circles). The resulting (*v*_*i*_, *v*_*j*_) pairs of mesh vertices are written to a binary file along with the corresponding lag time, Δ. For efficiency, all pairs are ordered so that the lower vertex index appears first (see for example the numbers highlighted in blue) and subsequently ordered along the first column, so that all particle displacements involving a given *v*_*i*_ appear as a single contiguous block. Finally, the VTP algorithm needs to be called on each source vertex *v*_*i*_ only once to evaluate all of the distances in the block. These evaluations are independent and therefore readily parallelized, as done in the current implementation. The computed displacements are assigned to both vertices while also taking account of the associated lag time.

To investigate the differences between the various diffusion measurement methods, we evaluated both the conventional Mean Square Displacement (by projecting the motion of the particles onto the macroscopic plane of the membrane) and the gMSD as presented above. The conventional and gMSD values as a function of the position along the *y* axis and the radial distance in the case of the “Wave” and “Budded” systems, respectively, are shown at a few selected lag times in Figure 3 and Figure 4.

**Figure 3:**
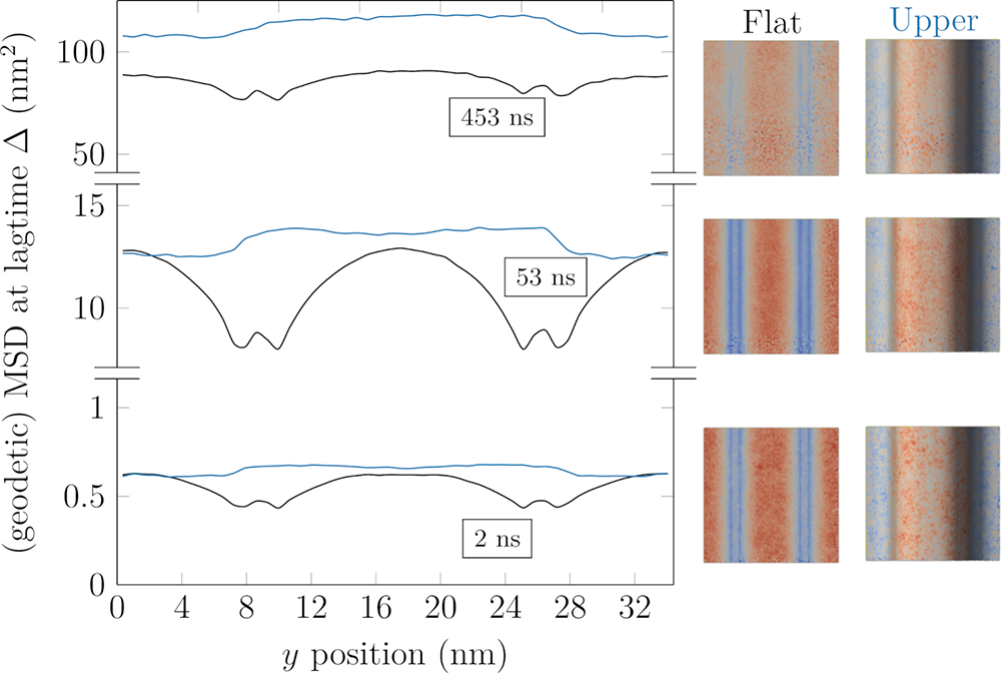
Two dimensional (“Flat”, discarded *z* coordinate, shown in black) and geodetic (“Upper”, for upper leaflet, shown in blue) MSD values at selected lag times (Δ = 2, 53, 453 ns from bottom upward) as a function of the position along the *y* axis in the “Wave” system. The values in the “Flat” system are averaged across both leaflets. The “Lower” leaflet is a shifted version of the “Upper” leaflet, and as such, it is omitted. The images on the right illustrate the distributions of MSD values at the corresponding lag times.

**Figure 4:**
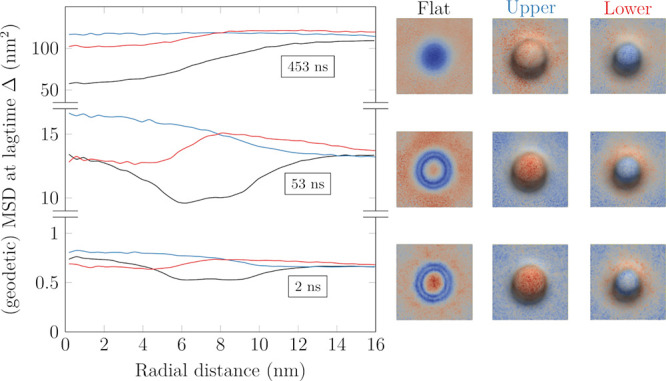
Two dimensional (“Flat”, discarded *z* coordinate, shown in black) and geodetic (“Upper” and “Lower” leaflet, shown in blue and red, respectively) MSD values at selected lag times (Δ = 2, 53, 453 ns from bottom upward) as a function of the distance from the center of the bud in the “Budded” system. The values in the “Flat” system are averaged across both leaflets. The images on the right illustrate the distributions of MSD values at the corresponding lag times.

In both systems, using the conventional MSD has a major effect on the apparent motion of the molecules due to ignoring their motion along the axis perpendicular to the plane of the membrane. The magnitude of this effect is directly related to local orientation of the membrane segments; thus, the projected displacements in the almost vertical regions of the “Wave” and “Budded” membranes underestimate the actual displacements the most. This phenomenon is purely geometric and can have a significant impact on results obtained from experiments. The conventional MSD profiles (Figures 3 and 4, black lines) get progressively smoother with increasing lag time due to the mixing of molecules originating from regions of different curvatures. Consequently, on infinite periodically repeating lattices such as the membranes simulated in the current study, the influence of curvature on the projected diffusion coefficients can be approximated in the long lag time limit as a simple geometrical scaling of the corresponding planar value,^28^ as discussed in the Supporting Information. In the “Budded” system (Figure 4), while at small lag times the center of the bud (*r* = 0) exhibits the largest conventional MSD values, this is completely obscured by the mixing with molecules originating from regions of lower apparent diffusion coefficient (the sides of the bud). Such a qualitative change in the apparent mobility of various regions has direct implications for the interpretation of experimental results on curved membranes, as it renders diffusion coefficients strongly time-dependent (as well as position-dependent).

When the conventional and the gMSD results are compared, it becomes clear that using the projected values manifests as an artificial slowdown on surfaces of both mean and Gaussian curvature, with a magnitude roughly proportional to the curvature. Hence, the use of geodetic displacements enabled by our tool is crucial for meaningful results, and when they are used, all MSD curves shift to higher values and become smoother as a function of spatial coordinates. In addition, similarly to the conventional MSD, molecules originating from regions of different curvature gradually mix as the lag time increases, producing uniform MSD profiles at large enough lag times. To better quantify these effects, we computed the diffusion coefficient distributions using the conventional yet incorrect MSD calculation method and the accurate geodesic-based gMSD approach presented here. Furthermore, we also decomposed the results from the latter based on local curvature (see the Supporting Information for details). The diffusion coefficients at the individual mesh points were calculated at an arbitrarily chosen lag time of 53 ns instead of separately applying linear regression to the MSD curves. It must be noted that computing a diffusion coefficient distribution on surface mesh points is not strictly equivalent to the per-particle distribution, as less frequently occupied mesh points should have a lower statistical weight. However, the almost uniform density of lipid centers of mass (see Figures S1 and S2) indicates that the distinction is insignificant. Indeed, the simple division using one lag time per mesh point MSD curve provided a very similar value to linear fits to MSD data calculated for lipids, validating our approach. The distributions of the diffusion coefficients in different membrane environments, shown in Figure 5, confirm that assuming 2D movement significantly underestimates the diffusion coefficient of molecules moving on curved surfaces (compare “Projected” and “Geodesic”). Additionally, while the flat region of the “Budded” system corresponds to the “apparently fastest” domain in the “Projected” distribution (both are around 6 × 10^–7^ cm^2^/s), the flat part of the “Wave” is conclusively faster than the projection. This is in agreement with the orientation of the planar membrane regions in the two systems (Figure 1).

**Figure 5:**
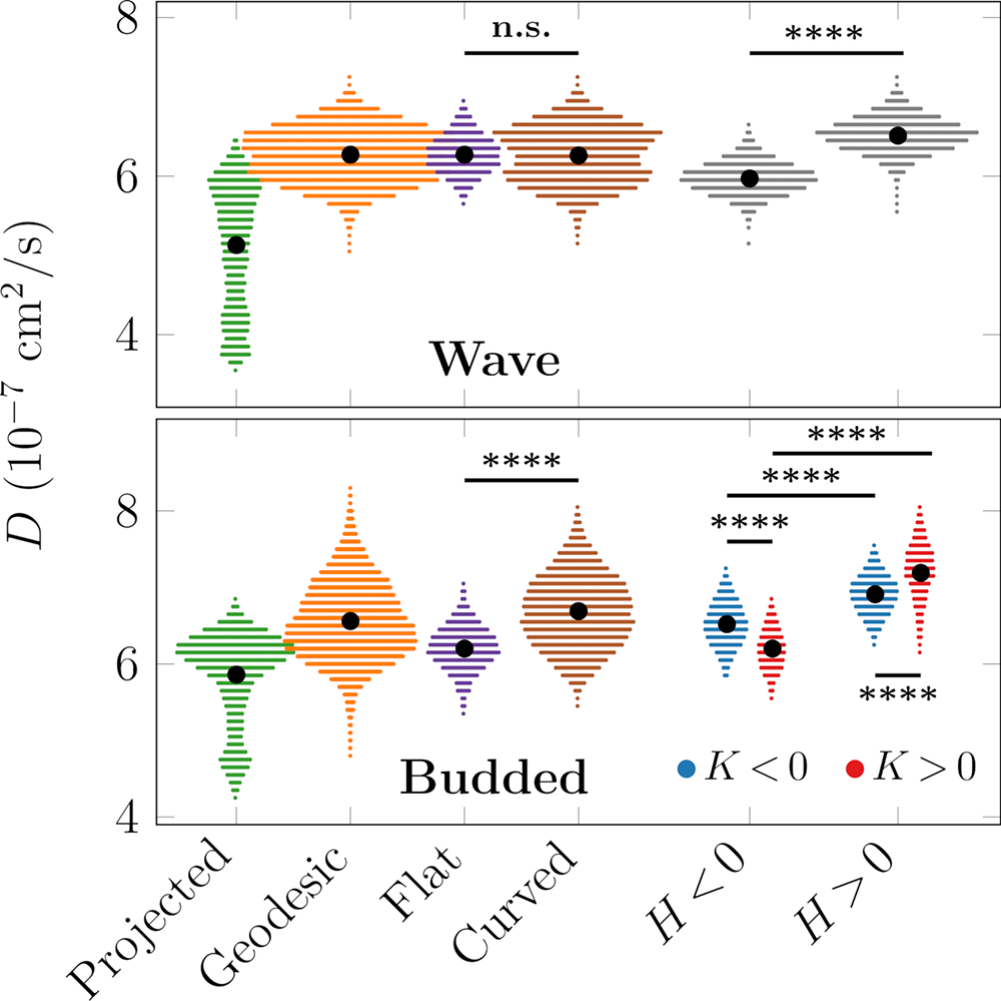
Spatial distributions of diffusion coefficients as determined by MSD/4Δ, Δ = 53 ns. Top: “Wave” system. Bottom: “Budded” system. The Projected and Geodesic coefficients were determined by the conventional 2D method and by using the geodetic distances, respectively. The latter values were further subdivided into categories based on their curvature (see the Supporting Information). *H*: mean curvature. *K*: Gaussian curvature. The black dots show mean values, and the area covered by the distributions is proportional to the prevalence of the corresponding curvature in the simulated system. Significance evaluated using the two-sample *t* test with unequal sample sizes. ****, *p* < 0.0001. “n.s.” stands for “not significant”.

Contrary to continuum theories predicting the lack of influence of mean curvature *H* on surface diffusion,^36−38^ the “Wave” system seems to exhibit clear correlations between the motion of particles and the mean curvature in regions with *H* > 0 and *H* < 0 based on Figure 3 and Figure S7. However, based on Figure 5, there is essentially no difference between the average diffusion coefficients on the flat and curved parts in the “Wave” system, thus leading to the apparent conclusion of *H* not affecting lateral diffusion. Nevertheless, by further decomposing the mean curvature into positively and negatively curved regions, we can conclude that *H* > 0 indeed enhances, while *H* < 0 hinders diffusion. These two effects are opposite and equal in magnitude, giving rise to zero net change compared to the flat domains.

The particles in regions of higher mean curvature are generally less densely packed (see the Supporting Information) in agreement with the results of Yesylevskyy et al.^17^ and hence more mobile. The converse is true for particles in regions of negative mean curvature, where the headgroups are more compressed.

Because there is a fundamental asymmetry in the curvature of its leaflets, the individual leaflets of the “Budded” system must be treated separately in the analysis. In the case of the upper leaflet (blue curves in Figure 4), positive Gaussian curvature, *K* > 0, seems to correlate with faster diffusion, while regions with *K* < 0 exhibit slower diffusion. This behavior is verified by the analysis in Figure 5 and goes against the conclusion drawn from continuum theories, where *K* > 0 results in slower diffusion and *K* < 0 causes faster diffusion.^37^ Similarly to the mean curvature, the speedup in the upper layer can be ascribed to a less dense packing of lipids, at least at the level of headgroups. This observed discrepancy between continuum predictions and molecular simulations highlights the importance of including lipid packing effects, which are usually not included in the continuum models^38^ and hence necessitate the development of tools to analyze particle-based simulations such as the present one. Even though the differences are minor in the systems studied here, they can be more significant with, e.g., a more complex lipid mixture in which the different lipid species are sorted by curvature.

Importantly, Gaussian curvature is insensitive to the direction of the membrane normal: bowls have positive and saddles have negative Gaussian curvature. Therefore, if the diffusion depended only on the Gaussian curvature, one would expect similar tendencies in both upper and lower leaflets. This is clearly not the case, as the lower leaflet seems to follow the theoretical prediction presented above.^37^ Consequently, the theoretically predicted role of Gaussian curvature is not the only factor determining surface diffusion in molecular systems; mean curvature also must play an important role. What is more, in our “Budded” system the absolute magnitude of Gaussian and mean curvatures positively correlate, while the mean curvature also encodes the orientation of the lipids. Thus, the mean curvature seems sufficient to explain the behavior observed in our simulations. Joined with the headgroup densities, the arising picture nicely follows that of the “Wave” system possessing only mean curvature. This line of reasoning is further supported by Figure S8 containing the correlations of the variables of interest (*H*, *K*, gMSD, and lipid headgroup densities).

To conclude, we have developed and optimized a novel algorithm and implemented it to analyze the diffusion dynamics along curved membrane surfaces. We have applied our method to two simulated membranes with different topologies: one with only mean curvature and another with additional Gaussian curvature. Our approach is the first one able to resolve the roles of membrane curvature and lipid packing on lipid diffusion. Our tool is readily applicable to multi-microsecond trajectories on systems spanning dozens of nanometers in size, i.e., to biologically relevant scales. This task is made possible only with the efficient implementation of the distance calculation in our algorithm. Our analysis method reveals rich details of lateral diffusion that are otherwise obscured by the use of conventional projected diffusion coefficients or continuum approaches. The present results not only shed light on the importance of lipid packing effects on the motion of the particles but also indicate the fundamental way by which the mean curvature *H* can affect diffusion. Based on the picture emerging following the analysis, either the mean curvature or the lateral density of lipid headgroups seems to be a reliable indicator of changes in lateral diffusion. Of course, the failure of the continuum models predicting the significance of Gaussian curvature *G* on the length scales of our simulated membranes is not unexpected, yet the starkness of the qualitative differences provides a sobering demonstration of the need to account for molecular level details; due to the high degree of curvature, the size of the lipid themselves becomes commensurate with the radius of curvature, increasing the relevance of lipid packing-related effects. The fundamentally different behavior of the upper and lower leaflets with respect to the Gaussian curvature has a significant conceptual impact on continuum theories^36,37^ and on simulations of elastic membrane models based on the Helfrich Hamiltonian.^2,45,46^ State-of-the-art simulation tools such as TriMem^47^ and FreeDTS^48^ are readily available for such simulations. Our approach presented here will not only help to better connect the (near) atomistic and mescoscopic simulations but will also aid in assigning local molecular properties—such as the diffusion coefficient, to the triangular faces.

## Data Availability

All simulation inputs and outputs are freely available at the Zenodo repository under the DOI-s listed in the Supporting Information. the implementation of the method is available at https://github.com/balazsfabian/curved-diffusion.

## Supporting Information Available

List of studied systems, details on the simulation methods and analyses; figures of MSD values vs distance from the center, vs position, vs lag time, in different Gaussian curvature regions, and in different “Wave” curvature regions (PDF)
