# Supplementary material for: Diffusion Analyses along Mean and Gaussian-Curved Membranes with CurD
Source: J Phys Chem Lett. 2024 Mar 14;15(11):3214–20. doi: 10.1021/acs.jpclett.4c00338 (PMC11444424; doi:10.1021/acs.jpclett.4c00338)
Supplement: Supplementary file 1 — jz4c00338_si_001.pdf [file jz4c00338_si_001.pdf]

Supporting Information: Diffusion Analyses Along Mean and Gaussian-curved Membranes with CurD

Balázs Fábián*,†,‡ and Matti Javanainen*,†,¶

†*Institute of Organic Chemistry and Biochemistry of the Czech Academy of Sciences, Flemingovo nám. 542/2, CZ-16000 Prague 6, Czech Republic*

‡*Current address: Department of Theoretical Biophysics, Max Planck Institute of Biophysics, Max-von-Laue Straße 3, 60438, Frankfurt am Main, Germany*

¶*Current address: Institute of Biotechnology, University of Helsinki, FI-00014 University of Helsinki, Finland*

E-mail: balazs.fabian@biophys.mpg.de; matti.javanainen@helsinki.fi

Contents

1 List of Studied Systems **S-2**

2 Details on Simulation Methods **S-3**

3 Details on Simulation Analyses **S-4**

3.1 Surface Meshing S-4

3.2 Lateral Density S-5

3.3 Conventional Mean Square Displacement S-6

3.4 Geodesic Mean Square Displacement S-8

3.5 Classification Based on Curvature S-10

3.6 Correlation of H, K, ρ_{hg} S-11

4 Additional Figures **S-13**

References **S-21**

1 List of Studied Systems

Table S1: Description of the coarse grained MARTINI systems simulated in this study. All membranes consisted of varying number of POPC (1-palmitoyl-2-oleoyl-sn-glycero-3-phosphocholine). The name "S" and "L" of the planar reference simulations indicate their sizes. The "Fixing method" refers to the approach used to keep the geometry of the membrane fixed, as discussed below.

System	Geometry	Fixing method	# Lipids	ZENODO DOI
L	flat	wall particles	6108	10.5281/zenodo.4643616
	flat	position restraints	6108	10.5281/zenodo.4643554
S	flat	wall particles	3642	10.5281/zenodo.4643634
	flat	position restraints	3642	10.5281/zenodo.4643632
Budded	curved	wall particles	4997	10.5281/zenodo.4196842
Wave	curved	wall particles	6108	10.5281/zenodo.4196842

2 Details on Simulation Methods

General Considerations A complete list of the simulated systems run in the Martini 2.2 coarse-grained force field[S1,S2] is provided in Table S1. All system were composed of neat, hydrated POPC bilayers, without any additional ions. Planar bilayers were generated using the `insane` (INSert membrANE) CG building tool.[S3] Curved systems were made using BUMPy.[S4] Notably, the Budded system contained 2546 lipids in the outer and 2451 lipids in the inner leaflet. To check for tension due to leaflet asymmetry, we computed the number of lipids in the flat rectangle spanned by the coordinates 0 to 6 nm in the x and y directions. The number of lower- and upper-leaflet lipids were 53.0±2.4 and 53.6±2.4, respectively, hinting at similar states of tension in the two leaflets. The target temperature of all the simulations was 310 K. Simulation parameters were chosen according to the recent suggested simulation settings ("New-RF").[S5] In addition, to keep the topological features from changing, the option `refcoord-scaling = no` was used. Therefore, while the lipid molecules belong to the NPT ensemble, the topological feature itself is treated as "NVT". For every system, 20 µs long trajectories were simulated using the Gromacs 2020.4 package.[S6,S7] All simulations are readily accessible under the DOIs provided in Table S1.

Fixing the Geometry Keeping large patches of lipid bilayers planar is commonly done by using a weak harmonic position restraints along the z axis of the simulation box.[S8] The restraining is applied only to ≈25% of the lipids of a single leaflet, thus enabling the equilibration of membrane thickness. In case of curved membranes, "wall" particles are added to the system.[S4,S9] These beads are weakly restrained in all three dimensions, and only interact with the acyl chains of the lipids. This approach was shown to only moderately perturb the structural properties of the membrane,[S9] while its effects of dynamics were not reported. In order to assess the effect on curvature on the surface diffusion of membrane lipids, we performed a set of planar simulations with the same composition as that of the curved systems. For the sake of consistency, we used "wall" particles to keep planar bilayers

from undulating.

Choosing a Reference Bead Another important issue is the box-size of the planar reference simulations: when comparing flat and curved system, is it the surface area or the projected area of the membrane that matters? As we are not aware of any general theory regarding this question, we simulated systems with the same number of lipids (that is, same surface area) and the same projected area to check its influence. The former are denoted by "S", while the latter with "L" indicating their sizes.

We acknowledge other possible sources of differences between the curved and flat membranes, such as finite-size effects[S10,S11] or assuming that the same form of the Oseen tensor holds for flat and curved bilayers.[S12] These effects were neglected in our comparisons.

3 Details on Simulation Analyses

3.1 Surface Meshing

Reference Surface Any kind of application of a continuum theory to bilayers requires a 2D surface. There are many possible choices such as the *pivotal plane*[S13,S14] or the *neutral plane*,[S15] but there is no theory that would unambiguously determine a single unique surface inside the membrane. Moreover, the pivotal plane is the location where no stretching or compression of the membrane happens, so the area per lipid matches the value of the flat bilayer.[S16] As such, it is not suited for surface density calculations. Here, we chose the surface formed by the center of mass of the individual leaflets. Similarly to the pivotal and neutral planes, the center of mass surface of the simulated POPC lipids also falls reasonably close to the glycerol backbones. However, its definition does not depend on the degree of curvature of the bilayer, so it can be used as a reference surface for MSD and also in principle for lateral density calculations. However, as discussed in the next paragraph, the results concerning the lateral density obtained using the center of mass reference surface are still not satisfactory.

3.2 Lateral Density

Lipid Center of Mass and Headgroup Density The lateral densities of the lipid centers of mass were evaluated in the simulated systems by assigning them to the closest mesh face, and dividing the resulting number of lipids by the area of the face. According to Yesylevskyy et al., the headgroup density decreases in regions of convex curvature and increases with concave curvature. The tendencies observed here in the "Wave" system (only mean curvature, Figure S1, solid curves) are completely in line with this notion. However, the picture emerging for the "Budded" system (non-zero Gaussian curvature, Figure S2, solid curves) cannot be rationalized in such a direct manner. For comparison, we repeated the analysis by constructing the mesh and calculating the density using the headgroup of the lipids. The results were completely in line with those originally found by Yesylevskyy et al. in both simulated systems, as demonstrated by the dashed curves in Figures S1 and S2. The smaller change in the center of mass densities compared to the headgroup densities is indicative of the fact that the former are closer to the pivotal plane, consequently are less sensitive to membrane deformations.

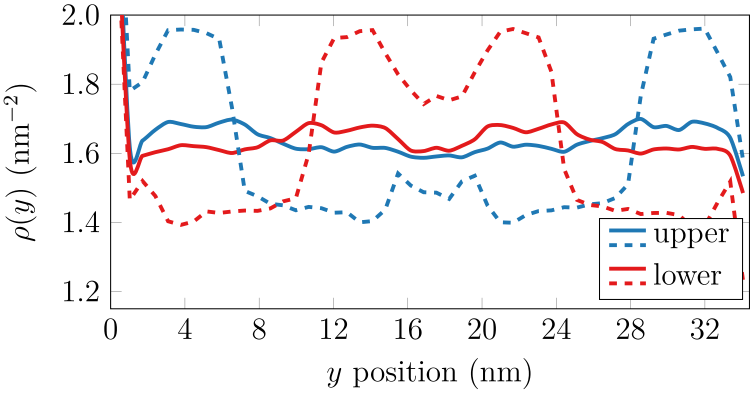

Figure S1: Lateral density of lipid centers of mass (solid) and lipid headgroups (dashed) as a function of the position along the y axis in the "Wave" system. Blue: upper leaflet, red: lower leaflet. Diverging values of density at the minimal and maximal positions are artefacts from the edges of the mesh.

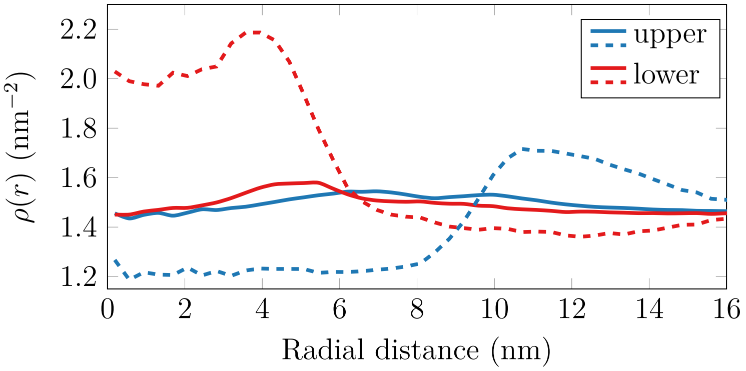

Figure S2: Lateral density of lipid centers of mass (solid) and lipid headgroups (dashed) as a function of the radial distance in the "Bud" system. Blue: upper leaflet, red: lower leaflet.

3.3 Conventional Mean Square Displacement

Center of Mass Removal The most common way to obtain reliable diffusion coefficients in simulations of planar membranes entails separating the upper and lower leaflets and calculating the Mean Squared Displacement (MSD) of lipids with respect to the center of mass of their host leaflets, thus eliminating any effects of the drift of the leaflet as a whole. This procedure was followed for the planar reference simulations. However, as the topological feature is kept constant during our simulations, removing the center of mass motion would result in nonzero translational motion of the topology. Any such motion would prevent meaningful calculation of diffusion on the surface. Fortunately, omitting the removal of the center of mass motion of the whole system did not result in substantial errors, and has no influence on the obtained results. The conventional MSD curves obtained from the simulations are presented in Figure S3.

Diffusion Coefficients The effect of curvature on lipid diffusion can be characterized as geometric and intrinsic, depending on its origin. The intrinsic effect is related to packing effects, and reflects changes in the local free energy of the lipid molecules. The geometric effect is simply the increased surface area of the surface on which the particle moves instead of the projected area that is computed from the lateral dimensions of the simulation box, or

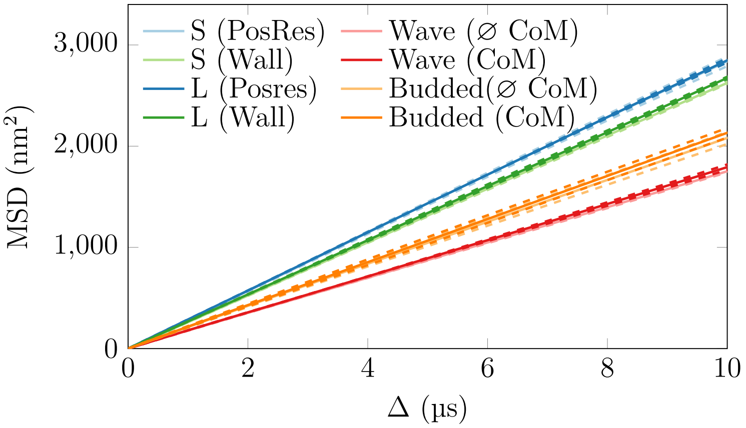

Figure S3: Mean Square Displacement (MSD) of POPC lipids in the simulated systems. "S" and "L" refer to the planar reference systems, "PosRes" and "Wall" are the methods to keep bilayer shape fixed. "Wave" and "Budded" are the two curved systems, for which the MSD curves were evaluated with and without removing the center of mass motion of the leaflet (∅ CoM, and CoM). Dashed curves are the MSDs of individual leaflets, while solid curves are the averages across both leaflets. Note that the large differences observed between the leaflets of the "Budded" system are due to the asymmetry and slightly different number of lipids between the upper and lower leaflet.

in our case, the topological feature. In the long lagtime limit, the geometric effect can be accounted for by a simple area scaling law, demonstrated by Naji and Brown,[S17] as

$$\left.\frac{D_{\mathrm{A}}}{D_0}\right|_{\text{area scaling}} = \frac{A_\perp}{A_{\mathrm{surf}}} \tag{1}$$

in 2 dimensions, or similarly

$$\left.\frac{D_{\mathrm{L}}}{D_0}\right|_{\text{area scaling}} = \left(\frac{L_\perp}{L_{\mathrm{c}}}\right)^2 \tag{2}$$

in 1 dimension. $A_\perp$ and $L_\perp$ are the projected surface area of edge length, and A_{surf} and L_{c} are the real surface area and line contour. D_{A} or D_{L} is the usual diffusion coefficient measured in the projected plane of the surface, while D_0 is the value after correcting for the increased area. The 1D expression is always exact, but the 2D is approximate for non-developable surfaces. Table S2 contains the diffusion coefficients obtained from the simulations. Note,

that even though the D_A of the “Wave” is smaller than that of the “Budded”, the case is quite the opposite when the diffusion coefficients are corrected by the surface area. Finally, while Naji and Brown found strong evidence in favor of the area scaling law in Equation 1, it seems to have a somewhat limited applicability to our simulations. Possible reasons for this are 1) not computing D_A at large enough lagtimes, 2) deviations due to non-flat free-energy landscape, for example due to lipid packing, 3) finite size effects.

Table S2: Diffusion coefficients in the simulated membranes. “S” and “L” refer to the planar reference systems, “PosRes” and “Wall” denote the methods to keep bilayer shape fixed. “Wave” and “Budded” refer to the two curved systems. All values are reported in units of [10^{-7} cm^2/s]. D_A refers to values obtained by performing linear fits to the conventional lateral MSD curves on the 1 µs to 10 µs range, while D_0 is the area corrected diffusion coefficient (see the text).

	L		S		Budded		Wave
	PosRes	Wall	PosRes	Wall	Upper	Lower	
D_A	7.18±0.01	6.70±0.02	7.09±0.05	6.52±0.03	5.25±0.01	5.54±0.01	4.49±0.03
D_0	-	-	-	-	6.21±0.01	6.35±0.01	6.91±0.03

3.4 Geodesic Mean Square Displacement

Periodic Boundary Conditions While the effect of PBC in conventional 2D or 3D MSD calculations can be trivially accounted for by simple vector addition, this relation no longer holds true on surfaces. To be able to calculate distances at lagtimes when the average displacement of the molecules is larger than the characteristic dimension of the simulation box, we create a surface mesh that covers (n_x, n_y) periodic images of the basic box. It can easily be seen that the use of a single periodic image with $n_x = n_y = 1$ is completely inadequate: the required minimum image positions of molecules passing through the boundaries of the basic box are not part of the mesh. Therefore, as a compromise between mesh size, discretization error and computational time, we use $n_x = n_y = 2$. In practice, this is not a major obstruction as long as one is only interested in the behavior of the MSD curves up to values corresponding to the dimensions of the topological feature, which completely contained in the basic simulation box.

Discretization Error Discretizing a molecular dynamics trajectory inherently comes with a certain loss of precision. The VTP (Vertex-oriented Triangle Propagation) algorithm[S18] provides a compromise between the continuous and the fully mesh-based distance computations: while similarly to Dijkstra's algorithm,[S19] the starting- and end-points of displacements are points the mesh, the path taken between these two vertices is not constrained to pass along the edges of the mesh. This difference is illustrated in Figure S4. To demonstrate

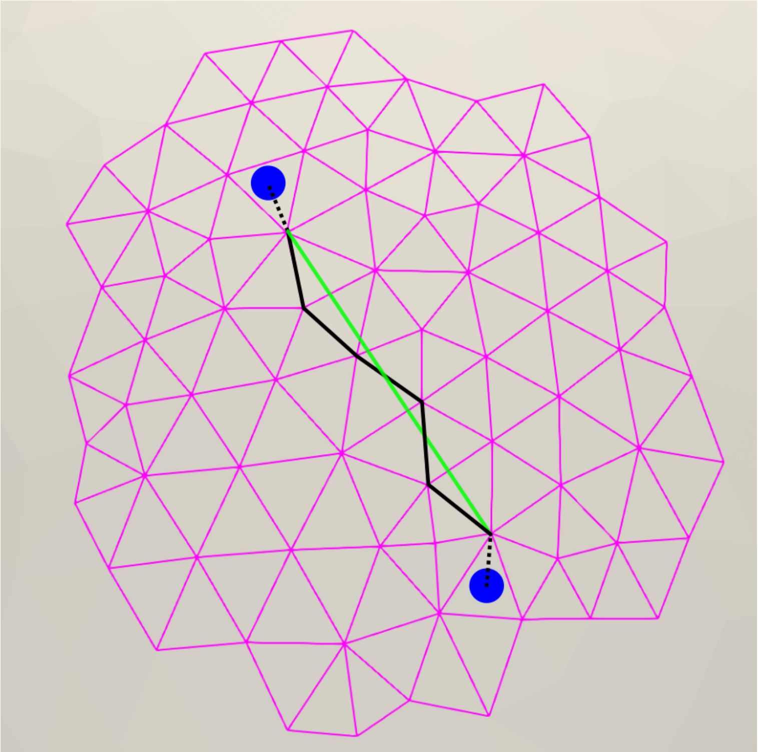

Figure S4: Possible discretization error in the geodesic MSD calculation scheme. Two distinct sources are (A) the discretization of the intial positions (B) the constraint to move along the edges. In the VTP algorithm, the geodesic curve is not restricted to pass along the edges of the mesh, therefore only affected by the first kind of error. (This figure was made using Paraview[S20])

the magnitude of errors incurred by using a discretized surface, we projected the "Wave" and "Budded" systems onto the xy plane, and evaluated the conventional 2D MSD curves on the resulting flat bilayers using the proposed method (based on the VTP algorithm[S18]) and `gmx msd` bundled with the GROMACS simulation package. As seen in Figure S5, besides the discretization error at short lagtimes, the two approaches are identical.

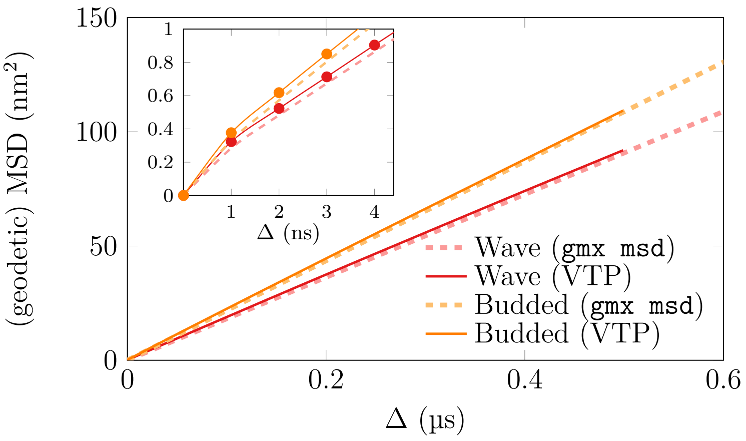

Figure S5: Comparison of the lateral MSD curves as computed by `gmx msd` and using the proposed method (denoted "VTP") on the 2D-projected version of the "Wave" and "Budded systems. The inset shows a magnification of the first few lagtimes. While the use of a discretized mesh results in differences at small lagtimes, at higher values two curves are essentially identical.

Scaling The scaling of the distance calculation algoritm (VTP[S18]) was investigated on meshes containing various number of points. For this, the same surface was represented by an increasing number of mesh points. The time required to evaluate all possible pairwise distances on the mesh ($t_{\text{single call}} \times n_{\text{points}}$) is presented in Figure S6. These timings are illustrative only of the VTP algorithm,[S18] and neglect the further processing of the resulting distances. Also, the values reflect the performance only on a single CPU (Intel(R) Core(TM) i7-7800X CPU @ 3.50GHz), while our implementation is embarrassingly parallel, due the independence of distance evaluations starting from different source vertices. For additional comparison, PyVista's[S21] implementation of the widely known Dijkstra's algorithm[S19] was found to be $\mathcal{O}(10^3)$ slower, and thus not included in Figure S6.

3.5 Classification Based on Curvature

In Figure 4 of the main text, we classify the diffusion coefficients calculated at the mesh points based on the local curvatures at these points. To separate flat and curved regions, we set a threshold values for K and H, as follows. We first histogrammed H or K, and

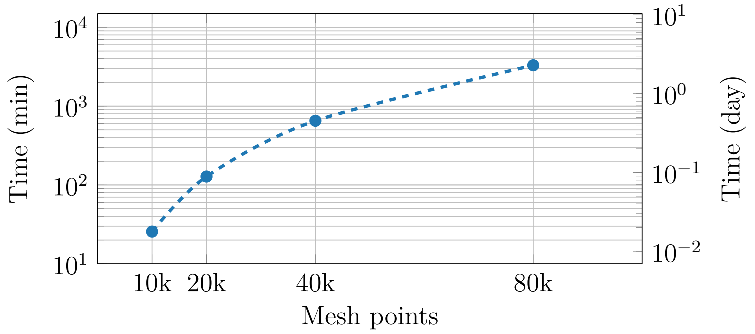

Figure S6: Time required to evaluate all pairwise distances on the meshed surface. The values are largely independent of the geometry of the mesh, and did not significantly change between the flat systems, the "Wave" and the "Budded". The standard deviations were less than 1%, and are omitted from the plot.

fit it with a multi-Gaussian function. The 95% confidence interval of the Gaussian function corresponding to zero curvature was extracted, and this interval was considered to correspond to zero curvature. We validated that the number of Gaussians in the fit had little effect on the outcome. The boundaries of the 95% confidence intervals for the Budded systems were ± 0.0016 nm^{-1} (H) and$\pm 3.7\times 10^{-6}$ nm^{-2}. For the Wave system, only H is present, and we found the boundaries of 0.003 nm^{-1}.

After the classification, the scatter column plot was generated by binning the D values into a histogram. A slight rounding the number of samples in each bin provided a symmetric figure, whereas every 20th point in each bin was shown.

3.6 Correlation of H, K, ρ_{hg}

To present a more detailed picture of the influence of the different types of curvature on the lateral mobility of the particles, we checked the correlations between the mean curvature (H), Gaussian curvature (K) and lipid headgroup densities (ρ_{hg}) in both the "Wave" and "Budded" systems. Just as in the above section, we based the analysis on the individual mesh points. The case of the "Wave" system is seen in Figure S7. Both the mean curvature (H) and the lipid headgroup density (ρ_{hg}) is a reliable indicator of the lateral mobility. The

former shows a clear correlation, while the latter exhibits anti-correlation with the mean curvature (H).

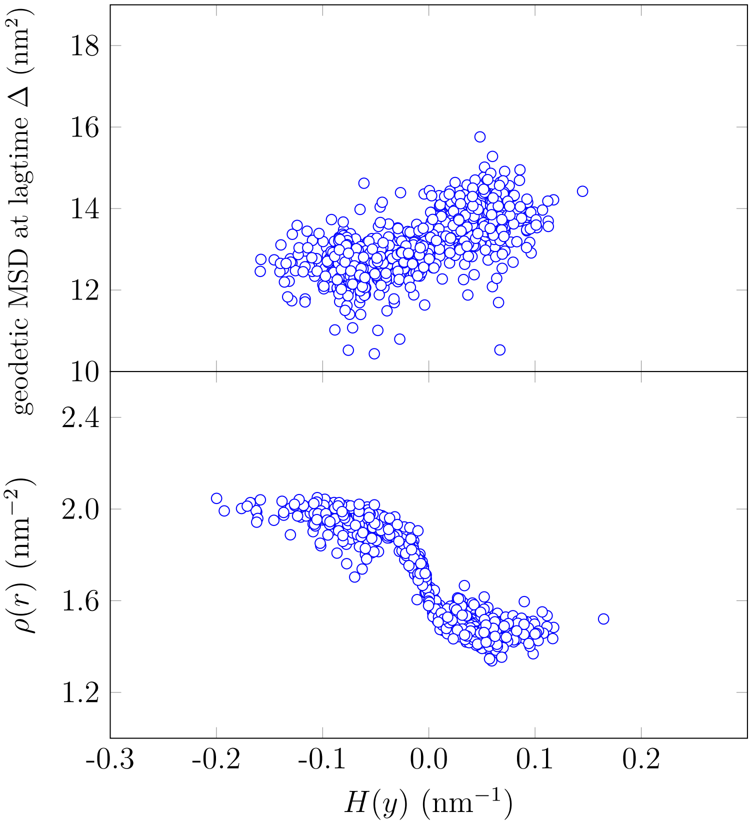

Figure S7: Correlation of the mean curvature in the "Wave" system with the geodesic MSD at Δ =53ns (TOP) and the lipid headgroup density (BOTTOM).

The correlations for the "Budded" system are presented in Figure S8. Clearly, the presence of Gaussian curvature along with the more complicated geometry of the bud makes both the correlations and the necessary plots more complex. Still, similarly to the "Wave", the mean curvature correlates nicely with the lateral mobility of the lipids, and anti-correlates with the headgroup density. However, the Gaussian curvature is not a reliable indicator, because it is insensitive to the orientation of the lipids in the bilayer, that is, whether the bud bulges into to extracellular or cytoplasmic space. The presence of a vertical "stalk" around zero mean curvature comes from the flat region of the membrane, and is the result of the intermixing of particles from differently curved regions with the flat domains.

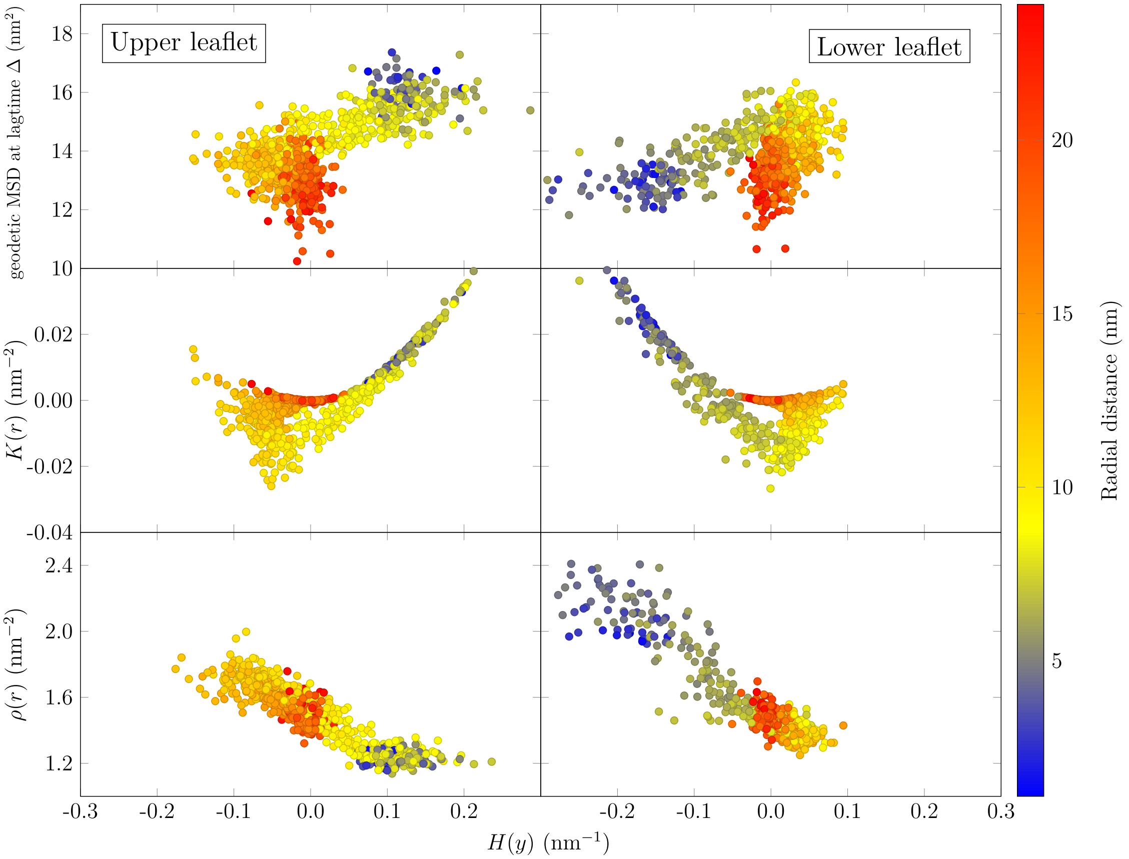

Figure S8: Correlation of the mean curvature in the "Wave" system with the geodesic MSD at Δ =53ns (TOP), Gaussian curvature (MID), and the lipid headgroup density (BOTTOM). The colors represent the radial distance as measured from the top of the bud. Because the upper and lower are not equivalent, they are presented separately, as indicated by the labels.

4 Additional Figures

Separate figures showing MSD curves at additional lagtimes (Δ) as a function of the position can be seen in Figures S9-S13, while the MSD curves averaged over distinct region of curvature are presented in Figures S14-S16.

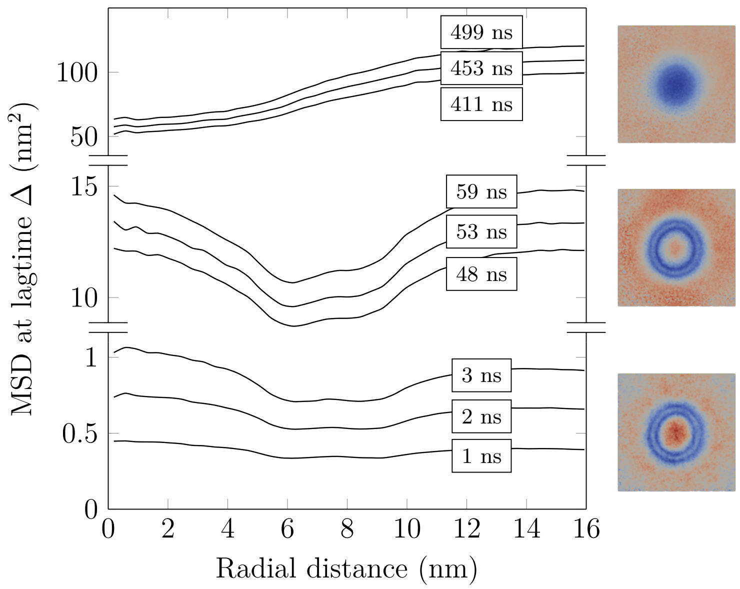

Figure S9: Two dimensional (discarded z coordinate) Mean Square Displacement values at selected lagtimes (Δ) as a function of the distance from the center of the bud in the "Budded" system. The values are averaged across both leaflets. The images on the right illustrate the distributions of MSD values at $\Delta = 2$, 53, 453 ns. At small lagtimes, the diffusion is fastest on the top of the bud, slower in the planar region. It is slowest on the "neck" of the bud as a result of projecting all displacements onto the xy plane. As the lagtime increases, a crossover happens due to the molecules of slower apparent diffusion reaching the top of the bud.

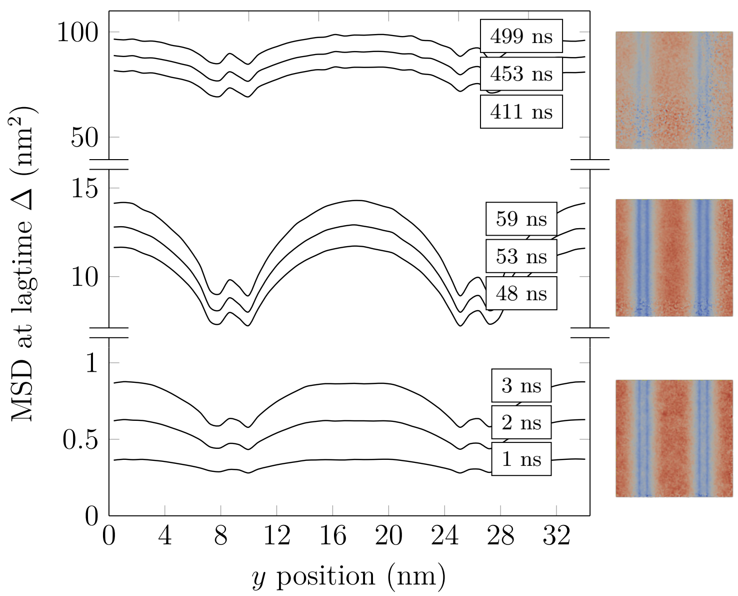

Figure S10: Two dimensional (discarded z coordinate) Mean Square Displacement values at selected lagtimes (Δ) as a function of the position along the y axis in the "Wave" system. The values are averaged across both leaflets. The images on the right illustrate the distributions of MSD values at $\Delta = 2$, 53, 453 ns. At all lagtimes, the diffusion is largest at the positions where the local membrane normal aligns with the axis of projection, z. The differences gradually disappear as Δ increases, due to the molecules of slower apparent diffusion penetrating into region where the apparent diffusion is faster.

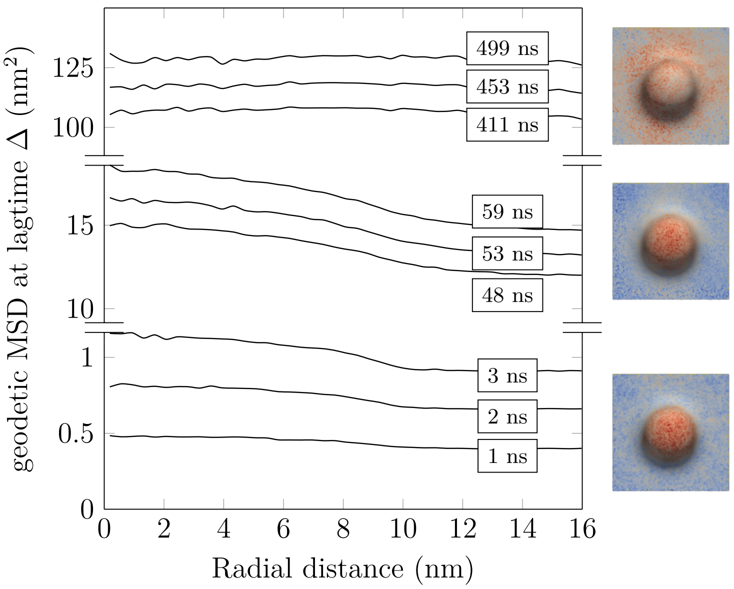

Figure S11: Geodesic Mean Square Displacement values at selected lagtimes (Δ) as a function of the distance from the center of the bud in the "Budded" system, upper leaflet. The images on the right illustrate the distributions of MSD values at Δ = 2, 53, 453 ns. The surface diffusion at all lagtimes is the fastest on the the top of the bud. The differences gradually disappear as Δ increases, due to the molecules of faster diffusion initially at the top of the bud reach the surrounding planar region.

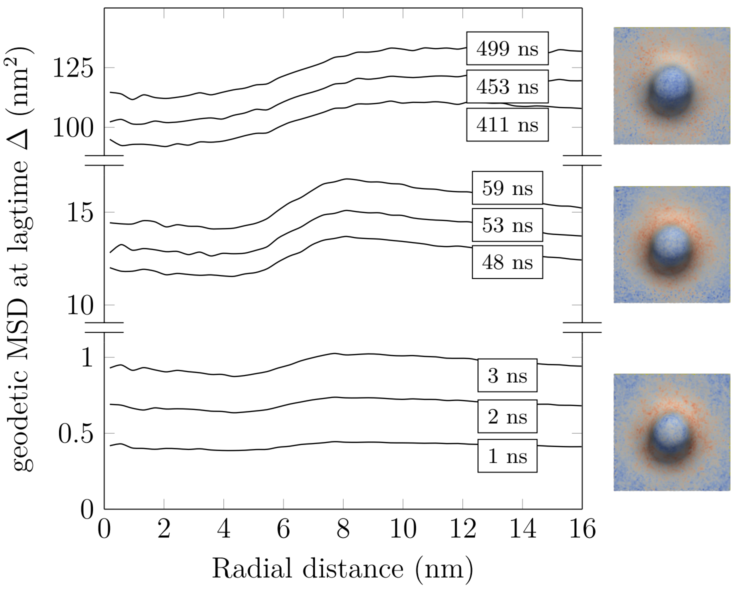

Figure S12: Geodesic Mean Square Displacement values at selected lagtimes (Δ) as a function of the distance from the center of the bud in the "Budded" system, lower leaflet. The images on the right illustrate the distributions of MSD values at $\Delta = 2$, 53, 453 ns. The surface diffusion at all lagtimes is the slowest on the the top of the bud. The differences gradually disappear as Δ increases, due to the molecules of faster diffusion initially in the planar region reach the top of the bud.

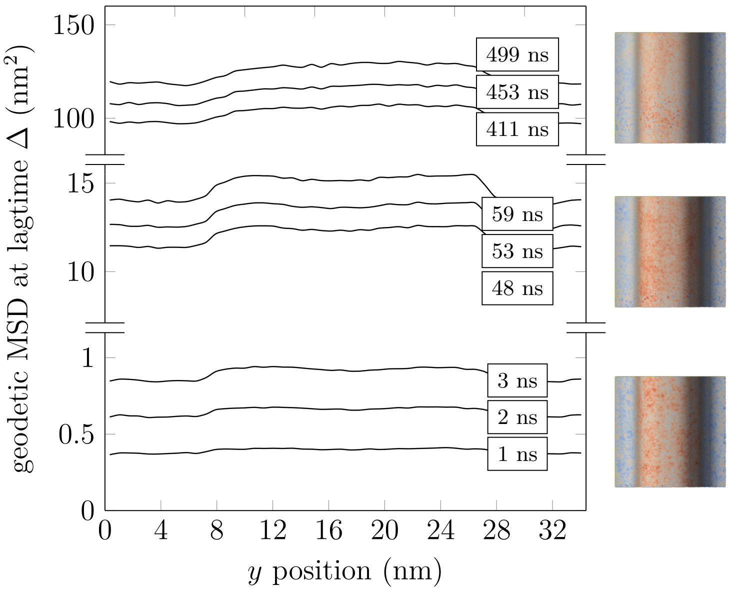

Figure S13: Geodesic Mean Square Displacement values at selected lagtimes (Δ) as a function of the position along the y axis in the "Wave" system, upper leaflet. The images on the right illustrate the distributions of MSD values at $\Delta = 2$, 53, 453 ns. At all lagtimes, the diffusion is largest at the positions where the leaflet has positive mean curvature, that is, it curves away from the headgroups. The differences gradually disappear as Δ increases, due to the exchange of molecules in the positively and negatively curved regions. Choosing the leaflet normal to point from the lipid tails towards the headgroups, the lower leaflet behaves the same way (not shown).

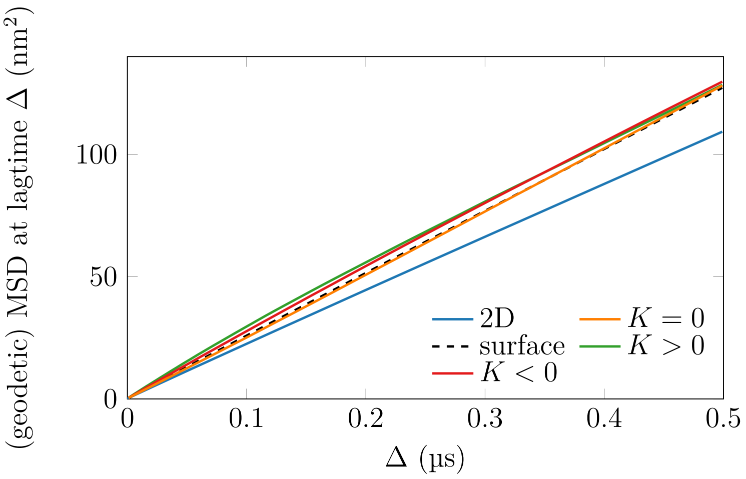

Figure S14: Mean Square Displacement in regions of different Gaussian curvature in the "Budded" system, upper leaflet. 2D (blue): conventional lateral MSD as obtained for the whole membrane, both leaflets, surface (black, dashed): overall geodesic MSD computed with the present method, $K > 0$ (green): geodesic MSD averaged over the region of positive Gaussian curvature, $K < 0$ (red): averaged over the region of negative Gaussian curvature, $K = 0$ (orange): averaged over the region of zero curvature.

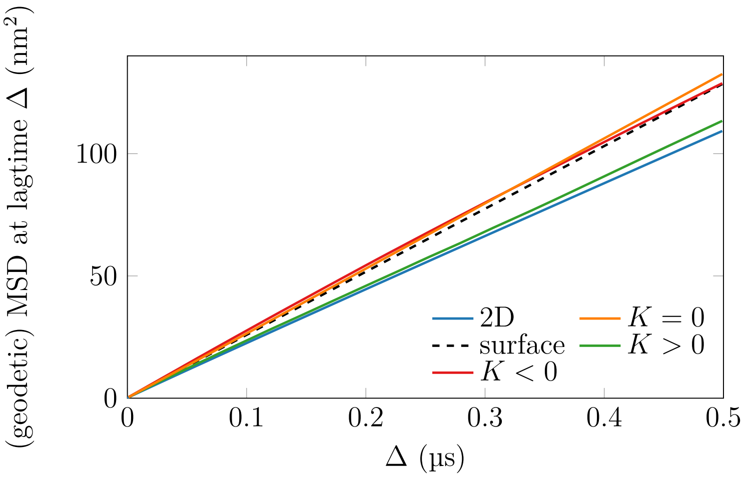

Figure S15: Mean Square Displacement in regions of different Gaussian curvature in the "Budded" system, lower leaflet. 2D (blue): conventional lateral MSD as obtained for the whole membrane, both leaflets, surface (black, dashed): overall geodesic MSD computed with the present method, $K > 0$ (green): geodesic MSD averaged over the region of positive Gaussian curvature, $K < 0$ (red): averaged over the region of negative Gaussian curvature, $K = 0$ (orange): averaged over the region of zero curvature.

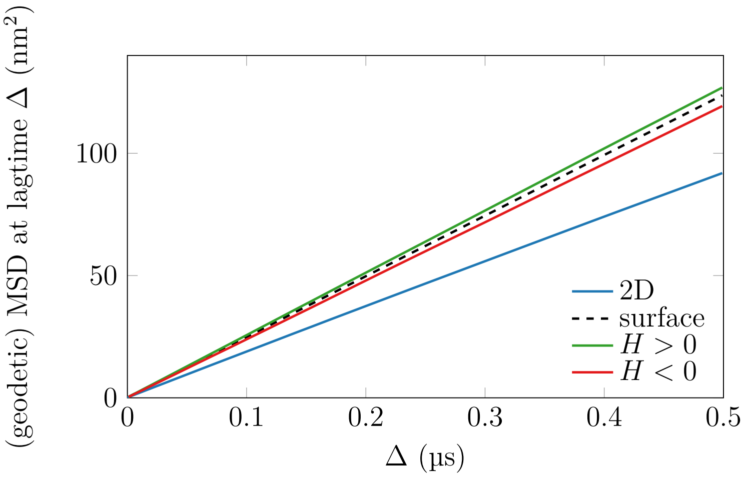

Figure S16: Mean Square Displacement in regions of different mean curvature in the “Wave” system, upper leaflet. 2D (blue): conventional lateral MSD as obtained for the whole membrane, both leaflets, surface (black, dashed): overall geodesic MSD computed with the present method, $H > 0$ (green): geodesic MSD averaged over the region of positive mean curvature, $H < 0$ (red): averaged over the region of negative mean curvature.

References

(S1) Marrink, S. J.; De Vries, A. H.; Mark, A. E. Coarse Grained Model for Semiquantitative Lipid Simulations. *J. Phys. Chem. B* **2004**, *108*, 750–760.

(S2) Marrink, S. J.; Risselada, H. J.; Yefimov, S.; Tieleman, D. P.; De Vries, A. H. The MARTINI Force Field: Coarse Grained Model for Biomolecular Simulations. *J. Phys. Chem. B* **2007**, *111*, 7812–7824.

(S3) Wassenaar, T. A.; Ingólfsson, H. I.; Böckmann, R. A.; Tieleman, D. P.; Marrink, S. J. Computational Lipidomics With *insane*: A Versatile Tool for Generating Custom Membranes for Molecular Simulation. *J. Chem. Theory Comput.* **2015**, *11*, 2144–2155.

(S4) Boyd, K. J.; May, E. R. BUMPy: A Model-Independent Tool for Constructing Lipid Bilayers of Varying Curvature and Composition. *J. Chem. Theory Comput.* **2018**, *14*, 6642–6652.

(S5) De Jong, D. H.; Baoukina, S.; Ingólfsson, H. I.; Marrink, S. J. Martini Straight: Boosting Performance Using a Shorter Cutoff and GPUs. *Comp. Phys. Commun.* **2016**, *199*, 1–7.

(S6) Abraham, M. J.; Murtola, T.; Schulz, R.; Páll, S.; Smith, J. C.; Hess, B.; Lindahl, E. GROMACS: High Performance Molecular Simulations Through Multi-Level Parallelism From Laptops to Supercomputers. *SoftwareX* **2015**, *1*, 19–25.

(S7) Páll, S.; Zhmurov, A.; Bauer, P.; Abraham, M.; Lundborg, M.; Gray, A.; Hess, B.; Lindahl, E. Heterogeneous Parallelization and Acceleration of Molecular Dynamics Simulations in GROMACS. *J. Chem. Phys.* **2020**, *153*, 134110.

(S8) Ingólfsson, H. I.; Melo, M. N.; Van Eerden, F. J.; Arnarez, C.; Lopez, C. A.; Wassenaar, T. A.; Periole, X.; De Vries, A. H.; Tieleman, D. P.; Marrink, S. J. Lipid Organization of the Plasma Membrane. *J. Am. Chem. Soc.* **2014**, *136*, 14554–14559.

(S9) Yesylevskyy, S. O.; Rivel, T.; Ramseyer, C. The Influence of Curvature on the Properties of the Plasma Membrane. Insights From Atomistic Molecular Dynamics Simulations. *Sci. Rep.* **2017**, *7*, 1–13.

(S10) Vögele, M.; Hummer, G. Divergent Diffusion Coefficients in Simulations of Fluids and Lipid Membranes. *J. Phys. Chem. B* **2016**, *120*, 8722–8732.

(S11) Vögele, M.; Köfinger, J.; Hummer, G. Hydrodynamics of Diffusion in Lipid Membrane Simulations. *Phys. Rev. Lett.* **2018**, *120*, 268104.

(S12) Camley, B. A.; Lerner, M. G.; Pastor, R. W.; Brown, F. L. Strong Influence of Periodic Boundary Conditions on Lateral Diffusion in Lipid Bilayer Membranes. *J. Chem. Phys.* **2015**, *143*, 12B604_1.

(S13) Rand, R.; Fuller, N.; Gruner, S.; Parsegian, V. Membrane Curvature, Lipid Segregation, and Structural Transitions for Phospholipids Under Dual-Solvent Stress. *Biochemistry* **1990**, *29*, 76–87.

(S14) Leikin, S.; Kozlov, M. M.; Fuller, N. L.; Rand, R. P. Measured Effects of Diacylglycerol on Structural and Elastic Properties of Phospholipid Membranes. *Biophys. J.* **1996**, *71*, 2623–2632.

(S15) Kozlov, M.; Winterhalter, M. Elastic Moduli for Strongly Curved Monoplayers. Position of the Neutral Surface. *J. Phys. II France* **1991**, *1*, 1077–1084.

(S16) Wang, X.; Deserno, M. Determining the Pivotal Plane of Fluid Lipid Membranes in Simulations. *J. Chem. Phys.* **2015**, *143*, 164109.

(S17) Naji, A.; Brown, F. L. Diffusion on Ruffled Membrane Surfaces. *J. Chem. Phys.* **2007**, *126*, 06B611.

(S18) Qin, Y.; Han, X.; Yu, H.; Yu, Y.; Zhang, J. Fast and Exact Discrete Geodesic Computation Based on Triangle-Oriented Wavefront Propagation. *ACM Trans. Graph.* **2016**, *35*, 1–13.

(S19) Dijkstra, E. W., et al. A Note on Two Problems in Connexion With Graphs. *Numer. Math.* **1959**, *1*, 269–271.

(S20) Ahrens, J.; Geveci, B.; Law, C. ParaView: An End-User Tool for Large Data Visualization. *The visualization handbook* **2005**, *717*.

(S21) Sullivan, C.; Kaszynski, A. PyVista: 3D Plotting and Mesh Analysis Through a Streamlined Interface for the Visualization Toolkit (Vtk). *J. Open Source Softw.* **2019**, *4*, 1450.
